# Mathematical model of SIR epidemic system (COVID-19) with fractional derivative: stability and numerical analysis

**DOI:** 10.1186/s13662-020-03192-w

**Published:** 2021-01-04

**Authors:** Rubayyi T. Alqahtani

**Affiliations:** Department of Mathematics and Statistics, College of Science, Imam Mohammad Ibn Saud Islamic University (IMSIU), Riyadh, Saudi Arabia

**Keywords:** SIR model, Stability, Nonlinear recovery rate, Hospital bed, Backward bifurcation, Fractional model

## Abstract

In this paper, we study and analyze the susceptible-infectious-removed (SIR) dynamics considering the effect of health system. We consider a general incidence rate function and the recovery rate as functions of the number of hospital beds. We prove the existence, uniqueness, and boundedness of the model. We investigate all possible steady-state solutions of the model and their stability. The analysis shows that the free steady state is locally stable when the basic reproduction number $R_{0}$ is less than unity and unstable when $R_{0} > 1$. The analysis shows that the phenomenon of backward bifurcation occurs when $R_{0}<1$. Then we investigate the model using the concept of fractional differential operator. Finally, we perform numerical simulations to illustrate the theoretical analysis and study the effect of the parameters on the model for various fractional orders.

## Introduction

The spread of Covid-19 diseases is a very complex phenomenon carried out by many researchers. Many mathematical models were proposed including complex and simple mathematical models to understand the disease behavior. Faal et al. [1] proposed a model for the spread of the COVID-19 disease taking into account the superspreader, hospitalized, and fatality class. The authors analyzed the local stability of the steady-state solution and the model sensitivity. Mandal et al. [2] introduced a mathematical model taking into account a quarantine class and governmental intervention measures. In this study, the authors consider the basic reproduction number as an important parameter in analyzing the dynamics of the model. Recently, significant works were carried out to study the behavior of COVID-19 by means of mathematical models. Lin et al. [3] proposed SEIR models for the COVID-19 using data from China considering the impact of social isolation policies including governmental actions. The model successfully captures the course of the COVID-19 outbreak, whereas Wells et al. [4] and Gostic et al. [5] consider the impact of travel restrictions and border control on the global spread of the COVID-19.

The SIR model is commonly used for disease modeling, in particular, for the COVID-19 analysis [6–8]. The dynamic behavior of SIR model, including the stability, bifurcation, and chaos, has been studied over many decades [9–12]. In most studies the authors assume that the recovery rate is a constant. However, in reality the recovery rate depends on time of recovering process such as the health system, including the number of hospital beds and medicines.

In recent years, many researchers have studied the systems of differential equations with fractional operators [13–15]. The epidemic models involving a fractional operator were also investigated by many authors because they deeply show biological and physical perspectives of the diseases [16, 17].

Rao et al. [18] studied an SIRS epidemic model assuming different death rates for each subclass, and the fraction of newborn children is represented by the parameter *p*. In this paper, we propose and analyze the extended SIRS epidemic model presented in [18] with the concept of fractional differential operator. In fact, we propose and study a model including three nonlinear differential equations with general incidence rate function and nonlinear recovery rate depending on the health system. The main focus of this study is analyzing the basic properties of model and demonstrating the stability properties of the model.

The rest of the paper is arranged as follows. We propose a dynamical model in Sect. 2. Then we formulate and establish the existence, uniqueness, positivity, and boundedness of solutions in Sect. 3. The steady-state solutions of the model and their stability are studied in Sects. 4 and 5, whereas numerical simulations of the steady-state solution brunches has is presented in Sect. 6. Section (7) contains a detailed dynamic behavior of the model with fractional derivative. We finish this study with conclusion in Sect. 8.

## The dimensional model

In this section, we extend the model suggested in [18] to include a nonlinear incidence rate and recovery rate. The recovery rate is a function of both the hospital bed-population ratio $b_{1} > 0$ and the infected I. Thus the recovery rate *α* is given by [19]

1 $$\begin{aligned} \alpha =\alpha _{0}+{ \frac{ ( \alpha _{1}-\alpha _{0} ) b_{1}}{\mathit{I}+b_{1}}}, \end{aligned}$$

where the parameter $\alpha _{1}$ and $\alpha _{0}$ are the maximum and minimum per capita recovery rates, respectively. The nonlinear incidence rate is generalized by the function

2 $$\begin{aligned} f(S,I)= \frac{\beta _{1} S I}{a_{1}+a_{2} S+a_{3} I}. \end{aligned}$$

Thus the system of differential equations is given by

3 $$\begin{aligned} &\frac{\mathit{dS}}{\mathit{dt}}=(1-p)b- \mu _{1}S -f(S,I)+\gamma R, \end{aligned}$$

4 $$\begin{aligned} &\frac{\mathit{dI}}{\mathit{dt}}=f(S,I)-(\mu _{2}+\alpha )I, \end{aligned}$$

5 $$\begin{aligned} &\frac{\mathit{dR}}{\mathit{dt}}=p b -(\mu _{3}+\gamma )R+ \alpha I, \end{aligned}$$

where the total population is split into three parts: $S(t)$ is the susceptible population, $I(t)$ is the infected population, and $R(t)$ is the recovered population, so that $N = S + I+ R$. The details and interpretation of the model can be found in [18]. We assume that all parameters are positive.

## Basic properties of model

### Positivity of solution

In this section, we prove that under nonnegative conditions, the model solutions are positive.

#### Theorem 1

*Let* $S_{0}, I_{0}, R_{0} \geq 0$. *The solution of* (3)*–*(5) *with* $(S(0), I(0),R(0)) = (S_{0}, I_{0}, R_{0})$ *is nonnegative*, *that is*, $S(t), I(t), R(t) \geq 0$ *for* $t > 0$.

#### Proof

Let $x(t) = (S(t), I(t), R(t))$ be the solution of system under initial conditions $x_{0} = (S(0), I(0), R(0)) = (S_{0}, I_{0}, R_{0})\geq 0$.

By the continuity of solution, for all of $S(t), I(t), R(t)$ that have positive initial values at $t = 0$, we have the existence of an interval $(0, t_{0})$ such that $S(t), I(t), R(t) \geq 0$ for $0 < t < t_{0}$. We will prove that $t_{0} = \infty $.

If $S(t_{1}) = 0$ for $t_{1}\geq 0$ and other solutions stay positive at $t = t_{1}$, then

6 $$\begin{aligned} \frac{\mathit{dS}}{\mathit{dt}}(t = t_{1}) = (1-p)A+\gamma R>0. \end{aligned}$$

This ensures that at any time the solution reaches the axis, its derivative increases, and the function $S(t)$ does not cross to negative part. We can show by similar analysis that

7 $$\begin{aligned} &\frac{\mathit{dI}}{\mathit{dt}}(t = t_{1}) =0, \end{aligned}$$

8 $$\begin{aligned} &\frac{\mathit{dR}}{\mathit{dt}}(t = t_{1}) =p b + \alpha I\geq 0. \end{aligned}$$

So $x(t)$ never crosses the axes $S = 0, I = 0, R = 0$ when it touches them. Thus, for any positive initial conditions, all equation solutions are positive. □

#### Theorem 1

*Let* ($S(t), I(t), R(t)$) *be the solution of system* (3)*–*(5) *with initial conditions* $(S_{0}, I_{0}, R_{0})$, *and let* $\mu = \min (\mu _{1}, \mu _{2}, \mu _{3} )$. *The compact set*

9 $$\begin{aligned} \Psi =& \bigl\{ \bigl(S(t), I(t), R(t)\bigr)\in {\mathbb{R}}^{3}_{+}, W \leq b/\mu \bigr\} \end{aligned}$$

*is positively invariant and attracts all solutions in* $\mathbb{R}^{3}_{+}$.

#### Proof

Let $W(t) = S (t)+ I (t) + R (t)$. Then from the system (3)–(5) we have

$$\begin{aligned} \frac{\mathit{dW}}{\mathit{dt}}\leq b-\min (\mu _{1}, \mu _{2}, \mu _{3} ) W =b-\mu W. \end{aligned}$$

This implies that

10 $$\begin{aligned} \frac{\mathit{dW}}{\mathit{dt}} + \mu W\leq & b. \end{aligned}$$

Solving (10), we obtain

11 $$\begin{aligned} 0< W \leq \frac{b}{\mu } +\biggl(W(0)- \frac{b}{\mu } \biggr) \exp (-\mu t), \end{aligned}$$

where $W(0)$ is the initial condition. Thus $0< W(t)<\frac{b}{\mu } $ as *t* reaches infinity, and hence Ψ is a positively invariant and attractive set. □

### Basic reproduction number

We use the next-generation matrix method [24] to calculate the reproduction number $R_{0}$ of model (3)–(5):

12 $$\begin{aligned} R_{0}={ \frac{ (\gamma _{1}+\mu _{3}[1-p] ) b\beta _{1}}{\mathit{a_{2}} (\gamma 1+\mu _{3}[1-p] ) ( \alpha _{1}+\mu _{2} ) b+{ \mathit{a_{1}}} \mu _{1} ( \mu _{3}+\gamma _{1} ) ( \alpha _{1}+\mu _{2} ) }}. \end{aligned}$$

## Equilibria

In this section, we consider the number of equilibrium solutions of model (3)–(5). It is clear that the model has a disease-free equilibrium given by

13 $$\begin{aligned} E_{0}(S,I,R)= \biggl( \frac{b (\gamma _{1}+\mu _{3}[1-p] ) }{\mu _{1} ( \mu _{3}+\gamma _{1} ) }, 0, \frac{p b}{\mu _{3}+\gamma _{1}} \biggr). \end{aligned}$$

The non-free steady state of model (3)–(5) can be obtained by setting the right sides to zero. From equations (3)–(5) we have

14 $$\begin{aligned} &S={ \frac{ ( \alpha _{0}+\mu _{2} ) {\mathit{I}}^{2}+ ( ( p-1 ) b+ b_{1}[\alpha _{1}+\mu _{2}]-\gamma _{1}R ) { \mathit{I}}+b b_{1} (p -1 ) -\gamma _{1} b_{1} R}{\mu _{1} ( \mathit{I}+\mathit{b_{1}} ) }}, \end{aligned}$$

15 $$\begin{aligned} &R= { \frac{\alpha _{0} {\mathit{I}}^{2}+ ( \mathit{b_{1}} \alpha _{1}+p b ) { \mathit{I}}+b \mathit{b_{1}} p}{ ( \mathit{I}+\mathit{b_{1}} ) ( \mu _{3}+ \gamma _{1} ) }}. \end{aligned}$$

Substituting equations ((14) and (15)) into equation (3), we obtain

16 $$\begin{aligned} E_{1}(I)=c_{3} I^{3} +c_{2} I^{2}+ c_{1} I + c_{0} =0, \end{aligned}$$

where $c_{0}$, $c_{1}$, $c_{2}$, and $c_{3}$ are defined by

17 $$\begin{aligned} &c_{3} = \bigl( ( \alpha _{0}+\mu _{2} ) \mu _{3}+ \gamma _{1} \mu _{2} \bigr) \beta _{1}+ ( \alpha _{0}+\mu _{2} ) ( \mathit{a_{2}} \alpha _{0}+ \mathit{a_{2}} \mu _{2}-\mathit{a_{3}} \mu _{1} ) \mu _{3} \\ &\phantom{c_{3} =}{} +\gamma _{1} ( \alpha _{0}+\mu _{2} ) ( \mathit{a_{2}} \mu _{2}-\mathit{a_{3}} \mu _{1} ), \\ &c_{2} = - \bigl( \gamma _{1}+\mu _{3}(1-p) \bigr) ( \mathit{a_{2}} \alpha _{0}+\mathit{a_{2}} \mu _{2}-\beta _{1} ) b+(c_{21}+c_{22}+c_{23})b_{1} +c_{24}, \\ &c_{1} =- \bigl( \gamma _{1}+\mu _{3}(1-p) \bigr) \bigl( a_{2}[ \alpha _{0}+\alpha _{1}+2 \mu _{2}]-2 \beta _{1} \bigr) \mathit{b_{1}} b +(c_{11}+c_{12}){\mathit{b_{1}}}^{2}+c_{13}, \\ &c_{0} = {\mathit{b_{1}}}^{2}[ R_{0}-1], \\ &c_{21}= \bigl( ( 2 \gamma _{1}+2 \mu _{3} ) {\mu _{2}}^{2} + ( \gamma _{1}+ 2 \mu _{3} ) ( \alpha _{0}+ \alpha _{1} ) \mu _{2}+2 \alpha _{0} \alpha _{1} \mu _{3} \bigr) \mathit{a_{2}}, \\ &c_{22}=- \bigl( 2 \mu _{1} \mu _{2} ( \mu _{3}+\gamma _{1} ) +\mu _{1} ( \mu _{3}+\gamma _{1} ) ( \alpha _{0}+ \alpha _{1} ) \bigr) \mathit{a_{3}}, \\ &c_{23}=- \bigl( 2\mu _{2} ( \gamma _{1}+ \mu _{3} )+ \mu _{3} ( \alpha _{0}+\alpha _{1} ) \bigr) \beta _{1}, \\ &c_{24}=-\mathit{a_{1}} \mu _{1} ( \mu _{3}+\gamma _{1} ) ( \alpha _{0}+\mu _{2} ), \\ &c_{11}= ( \alpha _{1}+\mu _{2} ) ( \mu _{3} \alpha _{1}+\gamma _{1} \mu _{2}+ \mu _{2} \mu _{3} ) \mathit{a_{2}}, \\ &c_{12}=- \bigl[ ( \mu _{3}+\gamma _{1} ) ( \alpha _{1}+ \mu _{2} ) \mu _{1} \mathit{a_{3}}+ ( \mu _{3} \alpha _{1}+ \gamma _{1} \mu _{2}+\mu _{2} \mu _{3} ) \beta _{1}\bigr], \\ &c_{13}=- ( \mu _{3}+\gamma _{1} ) ( \alpha _{0}+ \alpha _{1}+2 \mu _{2} ) \mathit{b_{1}} a_{1} \mu _{1}. \end{aligned}$$

If $R_{0}=1$, then $c_{0}=0$, so equation (16) reduces to the equation

18 $$\begin{aligned} E_{1}(I)=&I\bigl[a_{3} I^{2} +a_{2} I+ a_{1}\bigr] =0, \end{aligned}$$

where $I= 0$ is the disease-free equilibrium. By equation (16) the coefficient $c_{0} > 0$ when $R_{0} > 1$ and $c_{0} < 0$ when $R_{0} < 1$. Thus the number of possible positive real roots depends on the values of $c_{3}$, $c_{2}$, and $c_{1} $. The possible roots analyzed by the Descartes rule of signs are shown in Table 1.

**Table 1:** Number of possible positive real roots of equation (16). $c_{4}=$ basic reproduction number $R_{0}$, $c_{5}=$ sign change number, $c_{6}=$ possible number of positive real roots

Case	$c_{3}$	$c_{2}$	$c_{1}$	$c_{0}$	$c_{4} $	$c_{5} $	$c_{6}$
1	−	+	+	+	$R_{0}>1$	1	1
2	−	+	+	−	$R_{0}<1$	2	0, 2
3	−	+	−	+	$R_{0}>1$	3	1, 3
4	−	+	−	−	$R_{0}<1$	2	0, 2
5	−	−	+	+	$R_{0}>1$	1	1
6	−	−	+	−	$R_{0}<1$	2	0, 2
7	−	−	−	+	$R_{0}>1$	1	1
8	−	−	−	−	$R_{0}<1 $	0	0

### Theorem 2

*System* (3)*–*(5):

1. *has a one equilibrium if the basic reproduction number is greater than* 1 *and Cases* 1, 5, *and* 7 *are satisfied*;
2. *can have more than one equilibrium if the basic reproduction number is greater than* 1 *and Case* 3 *is satisfied*;
3. *can have two or more equilibria if the basic reproduction number is less than* 1 *and Cases* 2, 4, *and* 6 *are satisfied*.

The existence of multiple steady state suggests the possibility of backward bifurcation where the phenomenon of three branches of steady-state equilibrium occurs at the same point.

## Stability

In this section, we focus on analysis of the stability of the equilibrium of equations (3)–(5). We study the stabilities of two types of the disease equilibrium, that is, $E_{0}$ and $E_{1}$.

### Local stability of the disease-free equilibrium

In this section, we study the stability of the free equilibrium $E_{0}$. The Jacobian matrix of system (3)–(5) at $E_{0}$ is

19 $$ J(E_{0})= \begin{bmatrix} -\mu & j_{12} & \gamma _{1} \\ 0 & j_{22} & 0 \\ 0 & \alpha _{1} & -[\mu _{3}+\gamma _{1}] \end{bmatrix}, $$

where

$$\begin{aligned} &J_{12}=-{ \frac{\beta _{1} b ( \gamma _{1}+\mu 3_{1} [1-p] ) }{\mu _{1} ( \mu _{3}+\gamma _{1} ) } \biggl( { \frac{\mathit{a_{2}} b (\gamma _{1}+\mu _{3} [1-p] ) }{\mu _{1} ( \mu _{3}+\gamma _{1} ) }}+{ \mathit{a_{1}}} \biggr) ^{-1}}< 0, \\ &J_{22}=-{ \frac{ ( \gamma _{1}+\mu _{3}[1-p] ) ( \mathit{a_{2}} \alpha _{1}+\mathit{a_{2}} \mu _{2}-\beta _{1} ) b+\mathit{a_{1}} \mu 1 ( \mu _{3}+ \gamma _{1} ) ( \alpha _{1}+\mu _{2} ) }{\mathit{a_{2}} b (\gamma _{1}+\mu _{3}[1-p] ) +\mathit{a_{1}} \mu _{1} ( \mu _{3}+\gamma _{1} ) }}. \end{aligned}$$

The eigenvalues of matrix (19) are given by

20 $$ \lambda _{i}= \begin{bmatrix} -\mu _{1} \\ -[\mu _{3}+\gamma _{1}] \\ J_{22}\end{bmatrix}. $$

A simple calculation shows that $J_{22}=R_{0}-1$. So, we have the following result.

#### Lemma 1

*The free steady*-*state solution* $E_{0}$ *is locally asymptotically stable if* $R_{0} < 1$ *and is unstable if* $R_{0} > 1$.

### Stability of equilibria $E_{1}$

In this section, we show that the nonfree steady-state solution $E_{1}$ of system (3)–(5) is stable under specific condition. The Jacobian of the system can be written as

21 $$ J(E_{1})= \begin{bmatrix} -J_{11}& -J_{12}& \gamma _{1} \\ J_{21}& -J_{22} & 0 \\ 0 & J_{32} & -[\mu _{3}+\gamma _{1}] \end{bmatrix}, $$

where

22 $$\begin{aligned} &J_{11}= \biggl[{ \frac{\beta _{1} \mathit{I} ( \mathit{I} \mathit{a_{3}}+\mathit{a_{1}} ) }{ ( \mathit{I} \mathit{a_{3}}+\mathit{a_{2}} S+\mathit{a_{1}} ) ^{2 }}}+\mu _{1} \biggr], \end{aligned}$$

23 $$\begin{aligned} &J_{12}= \biggl[{ \frac{\beta _{1} S ( \mathit{a_{2}} S+\mathit{a_{1}} ) }{ ( \mathit{I} \mathit{a_{3}}+\mathit{a_{2}} S+\mathit{a_{1}} ) ^{2}}} \biggr], \\ &J_{21}= { \frac{\beta _{1} \mathit{I} ( \mathit{I} \mathit{a_{3}}+\mathit{a_{1}} ) }{ ( \mathit{I} \mathit{a_{3}}+\mathit{a_{2}} S+\mathit{a_{1}} ) ^{2}}}, \\ &J_{22}= \frac{ ( \alpha _{1}-\alpha _{0} ) {\mathit{b_{1}}}^{2}}{ ( \mathit{I}+\mathit{b_{1}} )^{2}}+ \alpha _{0}- { \frac{\beta _{1} S ( \mathit{a_{2}} S+\mathit{a_{1}} ) }{ ( \mathit{I} \mathit{a_{3}}+\mathit{a_{2}} S+\mathit{a_{1}} ) ^{2}}}+ \mu _{2}, \\ &J_{32}= \frac{ ( \alpha _{1}-\alpha _{0} ) {\mathit{b_{1}}}^{2}}{ ( \mathit{I}+\mathit{b_{1}} )^{2}}+ \alpha _{0}. \end{aligned}$$

From equation (4) we get the following relations:

24 $$\begin{aligned} &\frac{\beta _{1} S I}{a_{1}+a_{2} S+a_{3} I} -\biggl(\mu _{2}+\alpha _{0}+{ \frac{ ( \alpha _{1}-\alpha _{0} ) b_{1}}{\mathit{I}+b_{1}}}\biggr)I=0, \end{aligned}$$

25 $$\begin{aligned} &J_{22}= -{ \frac{\beta _{1} S\mathit{I} \mathit{a_{3}}}{ ( \mathit{I} \mathit{a_{3}}+\mathit{a_{2}} S+\mathit{a_{1}} ) ^{2}}}+{ \frac{ ( \alpha _{1}-\alpha _{0} ) \mathit{b_{1}} \mathit{I}}{ ( \mathit{I}+\mathit{b_{1}} ) ^{2}}}. \end{aligned}$$

By simple analysis we get that the characteristics equation of $J (E_{1})$ is

26 $$\begin{aligned} {\lambda }^{3}+ B_{1} {\lambda }^{2}+ B_{2} \lambda + B_{3}, \end{aligned}$$

where

$$\begin{aligned} &B_{1}=\mathit{J_{22}}+ \mathit{J_{11}}+\mu _{1}+\mu _{3}+\gamma _{1}, \\ &B_{2}= ( \mathit{J_{12}}+\mathit{J_{22}}+\gamma _{1}+\mu _{3} ) \mathit{J_{11}}+ ( { \mathit{J_{22}}}+\gamma _{1}+\mu _{3} ) \mu _{1}+ ( \gamma _{1}+\mu _{3} ) \mathit{J_{22}}, \\ &B_{3}= \bigl( ( \mathit{J_{12}}+\mathit{J_{22}}- \mathit{J_{32}} ) \gamma _{1}+\mu _{3} ( \mathit{J_{12}}+\mathit{J_{22}} ) \bigr) \mathit{J_{11}}+ J_{22}\mu _{1}[\gamma _{1}+\mu _{3}]. \end{aligned}$$

We further use the Rough–Hurtwiz criterion to show the stability of the steady state $E_{1}$. We have

27 $$\begin{aligned} \begin{aligned} &B_{1} B_{2}-B_{3}= B_{11}{ \mathit{J_{11}}}^{2}+ B_{22} {\mathit{J_{11}}}+ B_{33}, \\ &B_{11}= ( \mathit{J_{22}}+\mathit{J_{12}}+\mu _{3} + \gamma _{1} ), \\ &B_{22}= \bigl( {\mathit{J_{22}}}^{2}+ \bigl( \mathit{J_{12}}+ 2[ \mu _{3}+ \mu _{1}+ \gamma _{1} ] \bigr) \mathit{J_{22}}+{\gamma _{1}}^{2}+ \bigl( \mathit{J32}+2[ \mu _{3}+ \mu _{1}] \bigr) \gamma _{1}\\ &\phantom{B_{22}=}{}+\mu _{1} [{\mathit{J_{12}}}+2 \mu _{3} ]+{\mu _{3}}^{2} \bigr), \\ &B_{33}= ( \mu _{3}+\mu _{1}+\gamma _{1} ) {\mathit{J_{22}}}^{2}+ ( \mu _{3}+\mu _{1} +\gamma _{1} ) ^{2}{ \mathit{J_{22}}}+ \mu _{1} ( \mu _{3}+\mu _{1} +\gamma _{1} ) ( \mu _{3}+ \gamma _{1} ). \end{aligned} \end{aligned}$$

By the Routh–Hurwitz theorem $E_{1}$ is locally asymptotically stable when $B_{1} > 0$, $B_{3} > 0$, and $B_{1} B_{2}-B_{3} > 0$. Theses conditions are satisfied when the following condition holds:

28 $$\begin{aligned} { \frac{S }{ ( \mathit{I} \mathit{a_{3}}+\mathit{a_{2}} S+\mathit{a_{1}} ) ^{2}}}< \frac{\mu _{2}}{\beta _{1}}. \end{aligned}$$

Thus we have following results.

#### Lemma 2

*The steady*-*state solution* $E_{1}$ *of model* (3)*–*(5) *is locally asymptotically if*

29 $$\begin{aligned} { \frac{S }{ ( \mathit{I} \mathit{a_{3}}+\mathit{a_{2}} S+\mathit{a_{1}} ) ^{2}}}< \frac{\mu _{2}}{\beta _{1}}. \end{aligned}$$

#### Theorem 3

*The backward bifurcation occurs if* $b_{1}< b_{\mathit{cr}}$, *and no backward bifurcation otherwise*.

#### Proof

We show the conditions for the existence of backward bifurcation for system (3)–(5) using the center manifold approach.

First, making a transformation of variables, we have $x_{1} = S, x_{2} = I, x_{3} = R$. Then model (3)–(5) can be written in the form $\frac{\mathit{dX}}{\mathit{dt}} = F(X) $, where $F = ( f_{1}, f_{2}, f_{3})$. Hence

30 $$\begin{aligned} &\frac{\mathit{dS}}{\mathit{dt}}=f_{1}=(1-p)b- \mu _{1}S -f(S,I)+\gamma R, \end{aligned}$$

31 $$\begin{aligned} &\frac{\mathit{dI}}{\mathit{dt}}=f_{2}=f(S)-(\mu _{2}+\alpha )I, \end{aligned}$$

32 $$\begin{aligned} &\frac{\mathit{dR}}{\mathit{dt}}=f_{3}=p b -(\mu _{3}+\gamma )R+ \alpha I, \\ &\alpha = \alpha _{0}+{ \frac{ ( \alpha _{1}-\alpha _{0} ) b_{1}}{\mathit{I}+b_{1}}}, \\ &f(S,I)= \frac{\beta _{1} S I}{a_{1}+a_{2} S+a_{3} I}. \end{aligned}$$

Now let $\beta _{1}=\beta _{1}^{*}$ be the bifurcation parameter. When $R_{0}=1$, we have the following relation:

33 $$\begin{aligned} \beta _{1}=&{ \frac{\mathit{S_{0}} \mathit{a_{2}} [\alpha _{1}+\mu _{2}]+\mathit{a_{1}} [\alpha _{1}+\mu _{2}]}{\mathit{S_{0}}}}, \end{aligned}$$

and the model equation has one zero eigenvalue, and the other eigenvalues are negative. The behavior of the system near $\beta _{1}=\beta _{1}^{*}$ can be studied by applied the center manifold theory. The Jacobian matrix at free steady state $E_{0}$ is

34 $$\begin{aligned} J(E_{0})= \begin{bmatrix} -\mu _{1}&-{ \frac{\beta 1 \mathit{S_{0}}}{\mathit{S_{0}} \mathit{a_{2}}+\mathit{a_{1}}}}&\gamma 1 \\ 0&{ \frac{\beta _{1} { \mathit{S_{0}}}}{\mathit{S_{0}} \mathit{a_{2}}+\mathit{a_{1}}}}-\alpha _{1}-\mu _{2}&0 \\ 0&\alpha _{1}&-[\mu _{3}+\gamma _{1}] \end{bmatrix}. \end{aligned}$$

The right eigenvectors can be obtained as $W = (w_{1}, w_{2}, w_{3})^{T}$, where $(w_{1}, w_{2}, w_{3})^{T}=(-{ \frac{\alpha _{1} \mu _{3}+\mu _{2}[\gamma _{1}+\mu _{3}]}{\alpha _{1} \mu _{1}}}, {\frac{\mu _{3}+\gamma _{1}}{\alpha _{1}}}, 1 ) $. The left eigenvectors can be obtained as $V = (v_{1}, v_{2},v_{3}) = (0, 1, 0)$. The existence of backward bifurcation depends on the coefficients **a** and **b** in [25, Theorem 4.1]. The nonzero partial derivatives of system (30)–(32) at disease-free equilibrium $E_{0}$ are

35 $$\begin{aligned} &\frac{\partial f_{1}}{\partial x_{1}\, \partial x_{2}}(E_{0})=-{ \frac{ ( \alpha _{1}+\mu _{2} ) \mathit{a_{1}}}{\mathit{S_{0}} ( { \mathit{a_{2}}} \mathit{S_{0}}+\mathit{a_{1}} ) }}, \end{aligned}$$

36 $$\begin{aligned} &\frac{\partial f_{1}}{\partial x_{2}\, \partial x_{1}}(E_{0})=-{ \frac{ ( \alpha _{1}+\mu _{2} ) \mathit{a_{1}}}{\mathit{S_{0}} ( { \mathit{a_{2}}} \mathit{S_{0}}+\mathit{a_{1}} ) }}, \end{aligned}$$

37 $$\begin{aligned} &\frac{\partial f_{1}}{\partial x_{2}\, \partial x_{2}}(E_{0})= { \frac{\mathit{2 a_{3}} ( \alpha _{1}+\mu _{2} ) }{\mathit{a_{2}} \mathit{S_{0}} +\mathit{a_{1}}}}, \end{aligned}$$

38 $$\begin{aligned} &\frac{\partial f_{2}}{\partial x_{1}\, \partial x_{2}}(E_{0})={ \frac{ ( \alpha _{1}+\mu _{2} ) \mathit{a_{1}}}{\mathit{S_{0}} ( \mathit{a_{2}} \mathit{S_{0}}+\mathit{a_{1}} ) }}, \end{aligned}$$

39 $$\begin{aligned} &\frac{\partial f_{2}}{\partial x_{2}\, \partial x_{1}}(E_{0})={ \frac{ ( \alpha _{1}+\mu _{2} ) \mathit{a_{1}}}{\mathit{S_{0}} ( \mathit{a_{2}} \mathit{S_{0}}+\mathit{a_{1}} ) }}, \end{aligned}$$

40 $$\begin{aligned} &\frac{\partial f_{2}}{\partial x_{2}\, \partial x_{2}}(E_{0})=-2 \biggl( { \frac{ ( \alpha _{1}+\mu _{2} ) \mathit{a_{3}}}{\mathit{a_{2}} \mathit{S_{0}}+ \mathit{a_{1}}}}+ { \frac{\alpha _{0}-\alpha _{1}}{\mathit{b_{1}}}} \biggr), \end{aligned}$$

41 $$\begin{aligned} &\frac{\partial f_{3}}{\partial x_{2}\, \partial x_{2}}(E_{0})=2 \biggl( {\frac{\alpha _{0}-\alpha _{1}}{\mathit{b_{1}}}} \biggr). \end{aligned}$$

The coefficient **a** is obtained as

42 $$\begin{aligned} {\mathbf{a}} ={}&\sum_{k,i,j=1}^{3} v_{k} w_{i} w_{j} \frac{\partial f_{k}}{\partial x_{i}\, \partial x_{j}}= w_{1} w_{2} \frac{\partial f_{2}}{\partial x_{1}\, \partial x_{2}}(E_{0})+ w_{2} w_{1} \frac{\partial f_{2}}{\partial x_{2}\, \partial x_{1}}(E_{0}) + w_{2} w_{2} \frac{\partial f_{2}}{\partial x_{2}\, \partial x_{2}}(E_{0}) \\ ={}& {-}2 \biggl( { \frac{ a3 ( \mu _{3}+\gamma _{1} ) ^{2} ( \alpha _{1} +\mu _{2} ) }{{\alpha _{1}}^{2} ( \mathit{a_{2}} \mathit{S_{0}}+\mathit{a_{1}} ) }} + { \frac{ ( \alpha _{0}-\alpha _{1} ) ( \mu _{3}+\gamma _{1} ) ^{2}}{\mathit{b_{1}} {\alpha _{1}}^{2}}} \biggr) \\ &{}- { \frac{2 ( \alpha _{1} \mu _{3}+\mu _{2}[\gamma _{1}+ \mu _{3}] ) ( \mu _{3}+\gamma _{1} ) ( \alpha _{1}+\mu _{2} ) { \mathit{a_{1}}}}{{\alpha _{1}}^{2}\mu _{1} \mathit{S_{0}} ( \mathit{a_{2}} \mathit{S_{0}}+\mathit{a_{1}} ) }}. \end{aligned}$$

The bifurcation parameter **b** at $E_{0} $ is given by

$$\begin{aligned} \frac{\partial f_{2}}{\partial x_{2}\, \partial \beta _{1}^{*}}(E_{0})=&{ \frac{\mathit{S_{0}}}{\mathit{S_{0}} \mathit{a_{2}}+\mathit{a_{1}}}} \end{aligned}$$

and can be obtained as

43 $$\begin{aligned} {\mathbf{b}}& =\sum_{k,i=1}^{3} v_{k} w_{i} \frac{\partial f_{k}}{\partial x_{i}\, \partial \beta _{1}^{*}}= v_{2} w_{2} \frac{\partial f_{2}}{\partial x_{2}\, \partial \beta _{1}^{*}}(E_{0}) \\ &={ \frac{ ( \mu _{3}+\gamma _{1} ) \mathit{S_{0}}}{\alpha _{1} ( \mathit{S_{0}} \mathit{a_{2}}+\mathit{a_{1}} ) }} >0. \end{aligned}$$

Clearly, **b** is always positive. According to [25, Theorem 4.1], the backward bifurcation phenomenon exists when the coefficient **a** is positive. Thus the condition for backward bifurcation is given by

44 $$\begin{aligned} b_{1}< b_{1,\text{cr}}={ \frac{\mu _{1} \mathit{S_{0}} [ {\mathit{a_{2}}} ( \mu _{3}+\gamma _{1} ) ( \alpha _{1}-\alpha _{0} ) \mathit{S_{0}}+\mathit{a_{1}} ( \mu _{3}+\gamma _{1} ) ( \alpha _{1}-\alpha _{0} ) ] }{ [\alpha _{1}+\mu _{2}] [ { \mathit{S_{0}}}{\mathit{a_{3}}} \mu _{1} ( \mu _{3}+\gamma _{1} ) +\mathit{a_{1}} ( \alpha _{1} \mu _{3}+ \mu _{2}[\gamma _{1} +\mu _{3}] )] }}. \end{aligned}$$

 □

The existence of the backward bifurcation at $R_{0} = 1$ requires condition (44) to be satisfied. When the number of hospital beds $b_{1}$ is below the critical point $b_{1,\text{cr}}$, the number of hospital beds open to the public is below demand, and as a result, some patients fail to access to healthcare. In this situation, there remains a high infection leading to a backward bifurcation.

## Numerical simulations

**Figure 1:**
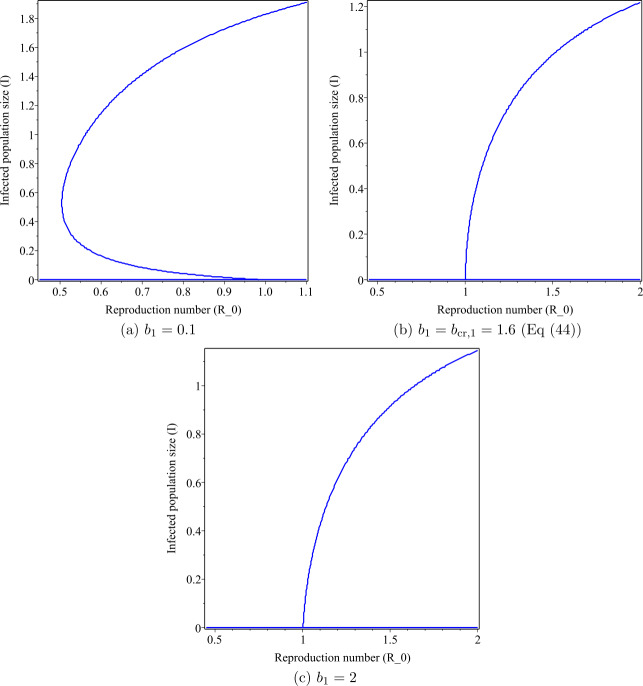
The figure showing a backward bifurcation varying the parameter $b_{1}$ for $R_{0} $. The values of the parameters are stated in Table 2

In this section, we carry out some numerical calculations to support our theoretical results. The values of parameters used for numerical simulations are indicated in Table 2. We study the branch of steady state with respect to the model parameters. Figure 1 shows the curves of the infected population *I* for different values of $b_{1}$, donated by the number of hospital beds and a specific value of general incidence rate ($a_{1}=a_{2}=a_{3}=1$). It shows that there is a forward bifurcation at $R_{0} = 1$.

**Table 2:** Parameters values

Parameters	Values	Reference
*b*	1	[20]
*p*	0.8 [0,1]	[21]
$\alpha _{0}$	0.0714	Assumed.
$\alpha _{1}$	0.0857	Assumed.
$\beta _{1}$	0.5	[20]
*γ*	0.25	[20]
$\mu _{1}$	0.2	[22]
$\mu _{2}$	0.2	[22]
$\mu _{3}$	0.2	[22]
$b_{1}$	[0,20] 1.9	[23]
$a_{1}$	1	Assumed.
$a_{2}$	1	Assumed.
$a_{3}$	1	Assumed.

**Figure 2:**
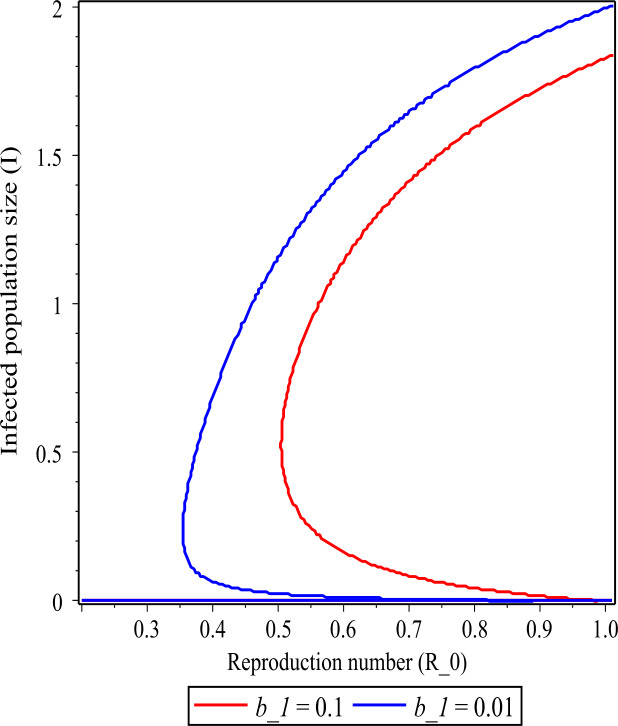
The figure showing a backward bifurcation varying the parameter $b_{1}$ for $R_{0} < 1$. The values of the parameters are stated in Table 2

If we decrease the value of $b_{1}$ from 2 to 1.6, then the backward bifurcation does not occur. These values are higher than the critical value of $b_{1,\text{cr}}=1.64$. If we decrease the value of $b_{1}$ to 0.1, less than the critical value $b_{1,\text{cr}}=1.64$, then we can observe from Fig. 1(a) that the backward bifurcation occurs. Note that in Fig. 1(a) the above line of the curve is a stable state and the below line of the curve is an unstable state. This result indicates that in managing an infectious disease the number of hospital beds plays a significant role. Figure 2 shows the effect of the value of $b_{1}$ on the curve when the backward bifurcation occurs. We observe that as the value of $b_{1}$ decreases, the area of the curve increases.

Figure 2 shows the infected population size *I* as a function of reproduction number $R_{0}$ when the parameter $b_{1}$ is varied for the case $R_{0}<1$. It illustrates that as the value of $b_{1}$ increases, the infected population size *I* decreases. It also shows the existence of a backward bifurcation, and the area of backward bifurcation curve decreases as the value $b_{1}$ increases.

## The model with fractional derivative

We consider the model with the Caputo–Fabrizio fractional derivatives

$$\begin{aligned} &D^{\alpha _{3}}_{t}S(t)=(1-p)b- \mu _{1}S -f(S,I)+\gamma R, \\ &D^{\alpha _{3}}_{t}I(t)=f(S)-(\mu _{2}+\alpha )I, \\ &D^{\alpha _{3}}_{t}R(t)=p b -(\mu _{3}+\gamma )R+ \alpha I, \\ &\alpha = \alpha _{0}+{ \frac{ ( \alpha _{1}-\alpha _{0} ) b_{1}}{\mathit{I}+b_{1}}}, \\ &f(S,I)= \frac{\beta _{1} S I}{a_{1}+a_{2} S+a_{3} I}. \end{aligned}$$

Here we have $0<\alpha _{3}<1 $ and

45 $$ D^{\alpha _{3}}_{t}=\frac{1}{\Gamma (1-\alpha _{3})} \int ^{t}_{0} f( \tau )' {(t-\tau )^{-\alpha _{3}}}\,d\tau. $$

We present the existence of positive solution of the system,

46 $$\begin{aligned} D^{\alpha _{3}}_{t}S(t)=&(1-p)b- \mu _{1} S -f(S,I)+\gamma R \geq - \mu _{1} S -f(S,I) \geq - \mu _{1} S. \end{aligned}$$

Then

$$\begin{aligned} S(t)\geq & S(0) \exp \bigl(-\mu _{1} t^{\alpha _{3}}\bigr) \quad\text{for all } t \in [0,t]. \end{aligned}$$

We can similarly show that

$$\begin{aligned} &I(t)\geq I(0) \exp \bigl(-(\mu _{2} +a_{0}) t^{\alpha _{3}}\bigr) \quad\text{for all } t \in [0,t]. \\ &R(t)\geq R(0) \exp \bigl(-(\mu _{3} +\gamma ) t^{\alpha _{3}}\bigr) \quad\text{for all } t \in [0,t]. \end{aligned}$$

Thus for all $t \in [ 0,t]$, we have that $S(t),I(t)$, and $R(t)$ are positive.

### Existence and uniqueness

Here we present the condition under which the system of equations has a unique solution. To achieve this, we have

47 $$\begin{aligned} &S(t)-S(0)=\frac{1}{\Gamma (\alpha _{3})} \int ^{t}_{0} f_{1}(S,I,R, \tau )_{1} {(t-\tau )^{\alpha _{3}-1}}\,d\tau. \end{aligned}$$

48 $$\begin{aligned} &I(t)-I(0)=\frac{1}{\Gamma (\alpha _{3})} \int ^{t}_{0} f_{2}(S,I,R, \tau )_{2} {(t-\tau )^{\alpha _{3}-1}}\,d\tau. \end{aligned}$$

49 $$\begin{aligned} &R(t)-R(0)=\frac{1}{\Gamma (\alpha _{3})} \int ^{t}_{0} f_{3}(S,I,R, \tau )_{3} {(t-\tau )^{\alpha _{3}-1}}\,d\tau. \end{aligned}$$

We will show that, for all $i=1,2,3$,

1. $| f_{i}( x_{i}, t)|^{2} \leq k_{i} (|x_{i}|^{2}+1)$ and
2. $| f_{i}( x_{i}, t)-f_{i}(x_{i}',t)|^{2} \leq k_{i} (|x_{i}-x_{i}'|^{2})$:

50 $$\begin{aligned} &\bigl\vert f_{1}(S,I,R,\tau ) \bigr\vert ^{2} \\ &\quad= \biggl\vert (1-p)b -\mu _{1}S - \frac{\beta S I}{a_{1}+a_{2}S+a_{3}I} +\gamma R \biggr\vert \end{aligned}$$

51 $$\begin{aligned} &\quad \leq 4\bigl((1-p)b \bigr)^{2} +4\mu _{1} \vert S \vert ^{2}+4\gamma ^{2} \vert R \vert ^{2}+ \frac{4\beta ^{2} \vert S \vert ^{2} \vert I \vert ^{2}}{ \vert a_{1}+a_{2}S+a_{3}I \vert ^{2}} \end{aligned}$$

52 $$\begin{aligned} &\quad\leq 4(1-p)^{2}b^{2} +4 \mu _{1}^{2} \vert S \vert ^{2} +4\gamma ^{2} \vert R \vert ^{2} + \frac{4\beta ^{2} \sup ( \vert S \vert ^{2} \vert I \vert ^{2})}{\min \vert a_{1}+a_{2}S+a_{3}I \vert ^{2}} \end{aligned}$$

53 $$\begin{aligned} &\quad\leq 4(1-p)^{2}b^{2} +4 \mu _{1}^{2} \vert S \vert ^{2} + 4\gamma ^{2} \Vert R \Vert _{\infty }^{2} +4\mu _{1}^{2} + \frac{4\beta ^{2} \Vert I \Vert ^{2}_{\infty }}{M} \vert S \vert ^{2} \end{aligned}$$

54 $$\begin{aligned} &\quad\leq \bigl(4(1-p)^{2}b^{2} +4 \mu _{1}^{2} \vert S \vert ^{2} + 4\gamma ^{2} \Vert R \Vert _{\infty }^{2} \bigr) \\ &\qquad{}\times \biggl( 1+ \frac{4\mu _{1}^{2} + \frac{4\beta ^{2} \Vert I \Vert ^{2}_{\infty }}{M} \vert S \vert ^{2}}{4(1-p)^{2}b^{2} +4 \mu _{1}^{2} \vert S \vert ^{2} + 4\gamma ^{2} \Vert R \Vert _{\infty }^{2}} \biggr) \end{aligned}$$

55 $$\begin{aligned} &\quad\leq \bigl(4(1-p)^{2}b^{2} +4 \mu _{1}^{2} \vert S \vert ^{2} + 4\gamma ^{2} \Vert R \Vert _{\infty }^{2} \bigr) \bigl( 1+ \vert S \vert ^{2} \bigr) \end{aligned}$$

56 $$\begin{aligned} &\quad\leq k_{1}\bigl( 1+ \vert S \vert ^{2} \bigr)\quad \text{if } \frac{4\mu _{1}^{2} +\frac{4\beta ^{2} \Vert I \Vert ^{2}_{\infty }}{M} }{4(1-p)^{2}b^{2} + 4\gamma ^{2} \Vert R \Vert _{\infty }^{2}}< 1, \end{aligned}$$

57 $$\begin{aligned} &\bigl\vert f_{1}(S,I,R,\tau )-f(S_{1},I,R,\tau ) \bigr\vert ^{2} \\ &\quad= \biggl\vert -\mu _{1}(S-S_{1}) - \frac{\beta (S-S_{1}) I}{a_{1}+a_{2}S+a_{3}I} \biggr\vert ^{2} \end{aligned}$$

58 $$\begin{aligned} &\quad\leq 2\mu _{1} S-S_{1} \vert ^{2} +2 \beta ^{2} \biggl\vert \frac{I}{a_{1}+a_{2}S+a_{3}I} \biggr\vert ^{2} \vert S-S_{1} \vert ^{2} \end{aligned}$$

59 $$\begin{aligned} &\quad\leq 2\mu _{1} \vert S-S_{1} \vert ^{2} +2 \beta ^{2} \biggl\vert \sup \frac{I}{a_{1}+a_{2}S+a_{3}I} \biggr\vert ^{2} \vert S-S_{1} \vert ^{2} \end{aligned}$$

60 $$\begin{aligned} &\quad\leq 2\mu _{1} \vert S-S_{1} \vert ^{2} +2 \beta ^{2} M \vert S-S_{1} \vert ^{2} \leq k_{2} \vert S-S_{1} \vert ^{2}, \end{aligned}$$

61 $$\begin{aligned} &\bigl\vert f_{2}(S,I,R,\tau ) \bigr\vert ^{2} \\ &\quad= \bigl\vert f(S,I)-(\mu _{2}+\alpha )I \bigr\vert ^{2} \end{aligned}$$

62 $$\begin{aligned} &\quad\leq \sup \bigl\vert f(S)-(\mu _{2}+\alpha ) \bigr\vert ^{2} \vert I \vert ^{2} \leq k_{3} \bigl(1+ \vert I \vert ^{2}\bigr), \end{aligned}$$

where

63 $$\begin{aligned} &k_{3}= \sup \bigl\vert f(S)-(\mu _{2}+\alpha ) \bigr\vert ^{2}, \end{aligned}$$

64 $$\begin{aligned} &\bigl\vert f_{2}(S,I,R,\tau )-f(S,I_{1},R,\tau )_{2} \bigr\vert ^{2} \leq k_{3} \vert I-I_{1} \vert ^{2} , \end{aligned}$$

65 $$\begin{aligned} &\bigl\vert f_{3}(S,I,R,\tau ) \bigr\vert ^{2} \\ &\quad = \bigl\vert p b -(\mu _{3}+\gamma )R+ \alpha I \bigr\vert ^{2} \end{aligned}$$

66 $$\begin{aligned} &\quad\leq 3 (p b)^{2} +3(\mu _{3}+\gamma )^{2} \vert R \vert ^{2}+ 3 \vert \alpha \vert ^{2} \vert I \vert ^{2} \end{aligned}$$

67 $$\begin{aligned} &\quad\leq 3 (p b)^{2} +3(\mu _{3}+\gamma )^{2} \vert R \vert ^{2}+ 3 \sup \vert \alpha \vert ^{2} \vert I \vert ^{2} \end{aligned}$$

68 $$\begin{aligned} &\quad\leq 3 (p b)^{2} +3(\mu _{3}+\gamma )^{2} \vert R \vert ^{2}+ M_{1} \end{aligned}$$

69 $$\begin{aligned} &\quad\leq 3\bigl( (p b)^{2} +M_{1}\bigr) (1+ \frac{(\mu _{3}+\gamma )^{2}}{(p b)^{2} +M_{1}} \vert R \vert ^{2} \end{aligned}$$

70 $$\begin{aligned} &\quad\leq 3\bigl( (p b)^{2} +M_{1}\bigr) \bigl(1+ \vert R \vert ^{2}\bigr)\quad \text{if } \frac{(\mu _{3}+\gamma )^{2}}{(p b)^{2} +M_{1}}< 1, \end{aligned}$$

71 $$\begin{aligned} &\bigl\vert f_{3}(S,I,R,\tau )-f_{3}(S,I,R_{3}, \tau )_{2} \bigr\vert ^{2}= (\mu _{3}+ \gamma )^{2} \vert R-R_{1} \vert ^{2} \leq k_{4} \vert R-R_{1} \vert ^{2}. \end{aligned}$$

Therefore, under the condition

72 $$\begin{aligned} \max \biggl( \frac{4\mu _{1}^{2} +\frac{4\beta ^{2} \Vert I \Vert ^{2}_{\infty }}{M} }{4(1-p)^{2}b^{2} + 4\gamma ^{2} \Vert R \Vert _{\infty }^{2}} , \frac{(\mu _{3}+\gamma )^{2}}{(p b)^{2} +M_{1}} \biggr)< 1, \end{aligned}$$

the system admits a unique solution.

### Numerical solution

In this section, we present the numerical solution of the equations. We use the numerical scheme of Atangan and Toufiq [26]. To use their scheme, we have

$$\begin{aligned} &D^{\alpha }_{t}S(t)=f_{1}( S,I,R,\tau ), \\ &D^{\alpha }_{t}I(t)=f_{2}( S,I,R,\tau ), \\ &D^{\alpha }_{t}R(t)=f_{3}( S,I,R,\tau ). \end{aligned}$$

The next step is converting the above to

73 $$\begin{aligned} &S(t)=S(0)+\frac{1}{\Gamma (\alpha _{3})} \int ^{t}_{0} f_{1}(S,I,R, \tau ) {(t- \tau )^{\alpha _{3}}}\,d\tau, \end{aligned}$$

74 $$\begin{aligned} &I(t)= I(0)+ \frac{1}{\Gamma (\alpha _{3})} \int ^{t}_{0} f_{2}(S,I,R, \tau ) {(t-\tau )^{\alpha _{3}}}\,d\tau, \end{aligned}$$

75 $$\begin{aligned} &R(t)=R(0)+\frac{1}{\Gamma (\alpha _{3})} \int ^{t}_{0} f_{3}(S,I,R, \tau ) {(t-\tau )^{\alpha _{3}}}\,d\tau. \end{aligned}$$

Following their scheme, we have

$$\begin{aligned} S(t_{n+1})={}&S(0)+\frac{(\triangle t)^{\alpha _{3}}}{\Gamma (\alpha _{3}+2)} \sum_{j=0}^{n} \bigl(f_{1}(S_{j},I_{j},R_{j},\tau _{j}) \bigr) (n+1-j )^{ \alpha _{3}} (n-j+2+\alpha _{3})\\ &{}-(n-j)^{\alpha _{3}}(n-j+1+\alpha _{3}) ) \\ &{}- \bigl(f_{1}(S_{j-1},I_{j-1},R_{j-1}, \tau _{j-1}) \bigr) (n+1-j )^{\alpha _{3}+1} -(n-j)^{\alpha _{3}}(n-j+1+ \alpha _{3}) ), \\ I(t_{n+1})={}&I(0)+\frac{(\triangle t)^{\alpha _{3}}}{\Gamma (\alpha _{3}+2)} \sum_{j=0}^{n} \bigl(f_{2}(S_{j},I_{j},R_{j},\tau _{j}) \bigr) (n+1-j )^{ \alpha _{3}} (n-j+2+\alpha _{3})\\ &{}-(n-j)^{\alpha _{3}}(n-j+1+\alpha _{3}) ) \\ &{}- \bigl(f_{2}(S_{j-1},I_{j-1},R_{j-1},\tau _{j-1}) \bigr) (n+1-j )^{\alpha _{3}+1} \\ &{}-(n-j)^{\alpha _{3}}(n-j+1+\alpha _{3}) ), \\ R(t_{n+1})={}&R(0) +\frac{(\triangle t)^{\alpha _{3}}}{\Gamma (\alpha _{3}+2)} \sum_{j=0}^{n} \bigl(f_{3}(S_{j},I_{j},R_{j},\tau _{j}) \bigr) (n+1-j )^{ \alpha _{3}} (n-j+2+\alpha _{3})\\ &{}-(n-j)^{\alpha _{3}}(n-j+1+\alpha _{3}) ) \\ &{}- \bigl(f_{3}(S_{j-1},I_{j-1},R_{j-1},\tau _{j-1}) \bigr) (n+1-j )^{\alpha _{3}+1} -(n-j)^{\alpha _{3}}(n-j+1+\alpha _{3}) ). \end{aligned}$$

**Figure 3:**
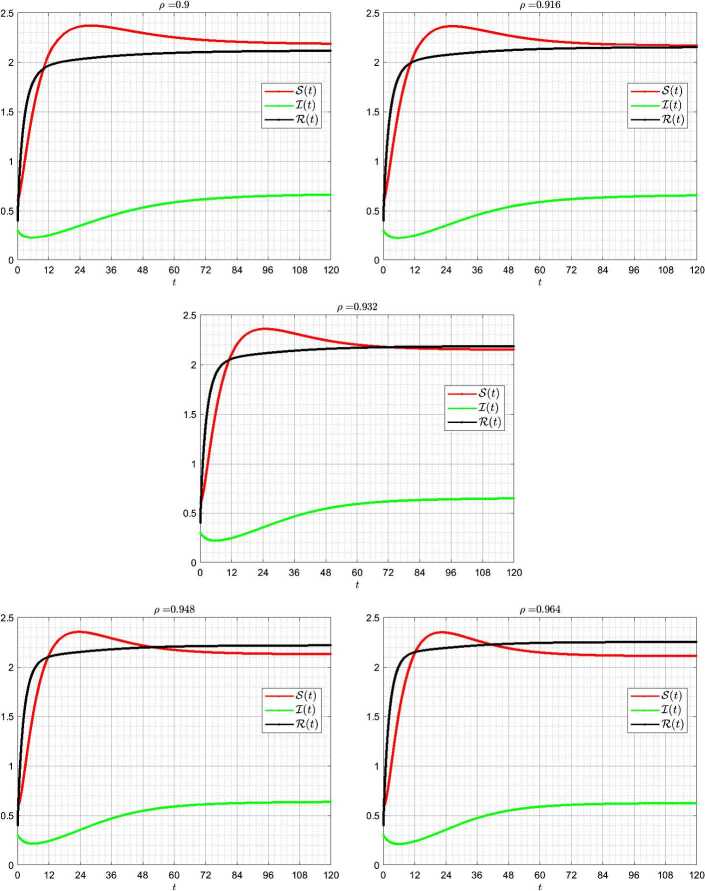
Numerical simulation of the susceptible population $S(t)$, the infected population $I(t)$, and the recovered population $R(t)$ for different values of fractional order. The values of the parameters are stated in Table 2

Figure 3 shows numerical simulations for different values of fractional order. We observe a slight change in the behavior of curves as the values of fractional order increase.

## Conclusion

In this paper, we considered the SIR model with general incidence rate function and nonlinear recovery rate to model the spread of disease. The nonlinear recovery rate depends on the influence of health system.

We proved the existence, uniqueness, and boundedness of the model solution. We studied all possible steady-state solutions of the model and details of stability and also derived the reproductive number. The analysis shows that the free steady state is locally stable when the reproductive number is less than unity and unstable otherwise. The model shows the phenomenon of backward bifurcation when $R_{0}<0$ and the parameter $b_{1}$ is less than the critical value given by

76 $$\begin{aligned} b_{1}< b_{1,\text{cr}}={ \frac{\mu _{1} \mathit{S_{0}} [ {\mathit{a_{2}}} ( \mu _{3}+\gamma _{1} ) ( \alpha _{1}-\alpha _{0} ) \mathit{S_{0}}+\mathit{a_{1}} ( \mu _{3}+\gamma _{1} ) ( \alpha _{1}-\alpha _{0} ) ] }{ [\alpha _{1}+\mu _{2}] [ { \mathit{S_{0}}}{\mathit{a_{3}}} \mu _{1} ( \mu _{3}+\gamma _{1} ) +\mathit{a_{1}} ( \alpha _{1} \mu _{3}+ \mu _{2}[\gamma _{1} +\mu _{3}] )] }}. \end{aligned}$$

When the parameter $b_{1}$ is sufficiently greater that the critical value $b_{1,\text{cr}}$, the disease infection decreases because the number of hospital beds increases. Therefore, to treat the disease in a community, the hospital resources must be improved.

Finally, we applied the theory of fractional derivatives to the model for different values of fractional orders. We used the numerical technique of Atangan and Toufiq, which is very accurate for solving fractional differential equations.

## Data Availability

Not applicable.
